# Ten simple rules for students navigating summer research experiences for undergraduates (REU) programs: From application to program completion

**DOI:** 10.1371/journal.pcbi.1011573

**Published:** 2023-11-09

**Authors:** Maria Manzanares, Courtney Peña, Kayla C. Kobak, Miranda B. Stratton

**Affiliations:** 1 Caltech, Student-Faculty Programs, Pasadena, California, United States of America; 2 Azusa Pacific University, Azusa, California, United States of America; 3 Stanford Biosciences Grant Writing Academy, Stanford University, Stanford, California, United States of America; 4 Office of Graduate Education, Stanford University, Stanford, California, United States of America; 5 Engineering Undergraduate Research Office, Purdue University, West Lafayette, Indiana, United States of America; 6 School of Medicine Human Resources Group Justice, Equity, Diversity, and Inclusion, Stanford University, Stanford, California, United States of America; Carnegie Mellon University, UNITED STATES

## Abstract

For many emerging scientists, research experiences for undergraduates (REU) programs are an important gateway to graduate school and a career in science, technology, engineering, and mathematics (STEM). REUs provide guided mentorship and learning experiences in a summer-long program where students develop research skills, build scientific knowledge, and strengthen their scientific identity. While the benefits of REUs are abundant, the process is not always easy to navigate, especially for students who come from first-generation and/or low-income (FLI) backgrounds. This paper provides two-fold guidance for undergraduate students interested in participating in REUs. Rules 1 to 5 focus on demystifying the application process from beginning to end, and Rules 6 to 10 guide students who are on the other side of the application process. Thus, this paper will be most helpful for undergraduate students who are either considering applying for an REU or have been accepted into one and want to learn more about what to expect. It can also be a shareable resource for faculty, staff, and mentors who work directly with STEM undergraduates.

This is a *PLOS Computational Biology* Methods paper.

## Introduction

Research experiences for undergraduates (REUs), also known as undergraduate research experiences (UREs), are an important part of an early scientist’s development [1–4]. These programs offer valuable opportunities for undergraduates to engage in hands-on scientific research during summer-long programs. Under the guidance of experienced research faculty and their trainees, REU’s provide authentic, practical learning experiences for students like you [5,6] as you develop essential research skills and gain a deeper understanding of the scientific inquiry process.

In addition to research, REUs often include professional development activities such as reading scientific papers, practicing poster presentations, preparing applications for graduate studies, and professional networking with peers and other scientists [3]. As an aspiring scientist, participating in an REU can serve as a prime steppingstone to science, technology, engineering, and mathematics (STEM) terminal degrees (such as a PhD or MD) [3,6–10]. REUs have also been found to offer affirming experiences for students as they consider future careers in science [6,11] and, notably, have been reported as a critical gateway for students from underrepresented/marginalized identity groups into STEM fields [1,3,5,7].

While the benefits of REUs are well-documented, we understand that navigating through the application, acceptance, and participation stages may require support and guidance. In this paper, we draw on both the existing research on REUs and our own experiences as leaders of summer REUs at Caltech, Stanford School of Medicine, and Purdue Engineering. Our aim is to offer 10 simple rules for navigating summer REUs, from the application stage through the completion of the program. This paper will be most useful if you are interested in applying for an REU (Rules 1 to 5) or preparing to participate in an upcoming summer REU program (Rules 6 to 10). The context of these rules is based on REU programs situated in the United States and may not be relevant for those outside of the US. Additionally, this paper can serve as a shareable resource for faculty, staff, and mentors who work with STEM undergraduates.

Throughout the paper, we refer to S1 File, the “REU Program Packet”, which is a downloadable and interactive resource we created for undergraduate students seeking REUs. The packet can be found in the Supporting information section of this paper.

## Rule 1: Explore your research interests

If you are interested in gaining research experience, not knowing where to begin can be overwhelming. Thankfully, numerous resources are available to get you started on the right track towards a STEM research career. Most undergraduate campuses have an undergraduate research office or career center that offers resources both for current students (if you want to stay at your home institution, aka the college or university where you are currently enrolled) or information for visiting undergraduates (for examples, see [12–15]). We also encourage you to explore resources like “Ten simple rules to make the most out of your undergraduate research career” [16] and “Ten simple rules for getting the most out of a summer laboratory internship” [17], which we will elaborate on in future rules like Rules 6 to 10. As you explore the research opportunities available to you, reflect on what scientific topics you are interested in and passionate about. Ask yourself the following questions:

- What excites you about science?
- Why do you want to do research?
- What scientific topics capture your interest? These could be topics you have learned about in courses, topics you have heard or read about in the news, or based on your life experience (e.g., you know someone affected by a particular disease).
- What kind of impact do you want your research to have? For example, are you eager to find a cure for a disease or to uncover the inner workings of a natural phenomenon?
- What scientific topics are less exciting or interesting to you? Why?
- If you have had previous research experience, what have you enjoyed/not enjoyed about your experience?

Reflecting on these questions will help you narrow down your research interests and identify the most relevant research topics and REUs for you. However, keep in mind that your journey towards research may not follow a linear path. It’s crucial to keep an open mind and be willing to explore different areas. Engaging in research experiences, especially if you are new to the field, can help you discover the scientific domains that truly resonate with your interests and passions. Seeking guidance from mentors and experienced researchers is invaluable during this exploratory phase. Remember, it’s okay not to know everything at this stage—lean on those who have been where you are to learn about how they got there and see what you can learn from their journey. Once you have reflected on the kind of research you are interested in Rule 1, utilize pages 1 to 4 of S1 File to help you further identify and narrow down your motivations.

## Rule 2: Identify your programmatic needs

As you explore potential programs, think about what you would like to get out of your summer research experience and what will help you become a successful scientist. Your scientific identity is one facet of your multidimensional self, and you can nurture it alongside your other social identities. Particularly, personal financial goals and considerations are crucial to the process of choosing an REU. Meeting your basic needs, such as food and housing security, is integral to success in any endeavor. Meeting basic needs may particularly be a concern if you are a student whose identities are underrepresented in STEM or for first-generation/low-income (FLI) students [18]. To determine what considerations are important for you, we recommend asking yourself the following questions:

- What is my availability, given my academic calendar?
- What are my financial and housing needs?
- Am I open to fully remote, hybrid, or in-person programs?
  ○ If pursuing a hybrid or in-person experience, will I need funding and logistical support for travel, housing, meals, and other needs?
  ○ If pursuing a remote experience, do I have the resources necessary to participate and reach my fullest potential (e.g., stable internet, a computer with high computing power, etc.)?
- Can I leave my current living situation and shift my responsibilities to pursue an opportunity (e.g., apartment leases, job and/or family responsibilities)?
- What are my accessibility-related needs (if any)? Think about what accommodations you currently have at your home institution and what needs still need to be met. This could be related to visible [19] and invisible disabilities [20], as well as temporary or acquired disabilities (for more information, see [21]).
- What additional resources will I need to be successful?

Interests (Rule 1) and needs (Rule 2) are both important in determining which programs will be your best fit (Rule 3) as well as in the creation of your application materials (Rule 4), so be sure to spend some time on this groundwork before moving forward.

## Rule 3: Find your programmatic fit

Now that you better understand your interests and needs for the summer, you can start exploring different programs and evaluating how they align with what you hope to gain from the experience. When looking for research programs, use tools such as the National Science Foundation’s REU search tool [22] and the American Association of Medical College’s Summer Undergraduate Research Programs [23] to identify programs by field, keywords, or location. You can also search for programs at schools of interest and use tools like Pathways to Science [24]. You may also search at government labs and/or companies in industry.

If you are an international or undocumented student, please note that some programs have resident restrictions for funding purposes, but many do not. You will need to check the specific requirements of each program. DACA students can find various opportunities through resources such as Summer Research Programs in STEM for Undocumented and DACA Students [25] and Programs for Undocumented Students [26]. Peruse the website and connect with office staff to ask questions.

Additionally, reach out to your academic networks including faculty, advisors, and staff to identify what research opportunities exist and when to consider applying for them in your undergraduate career. Conversations with them can be informative and may even result in potential connections. If possible, talk to at least 3 people to have multiple perspectives. These conversations can also be helpful in case your REU application requires a reference letter. Similarly, leverage your peers and friends for program recommendations if they have participated in REUs or have heard about them from conferences, LinkedIn, or from their support networks.

You can even directly contact the faculty you are interested in conducting research with. Some programs will require this as a first step, but many do not. Usually, first contact consists of an email, but you can also suggest an informational interview where you can mention the program you are applying to at their institution and ask about their experience with it. Don’t be afraid to reach out to your faculty networks to facilitate connections with faculty at other institutions: word-of-mouth referrals can go a long way in helping to achieve your goals.

## Rule 4: Create an application timeline and materials checklist

Once you have identified which program(s) you are most interested in, use the REU program’s website to collect information on the application process. If you have any outstanding questions regarding needs you identified in Rule 2 that are not addressed on the program’s website, contact program staff for more information. Typically, the program staff is available to address specific questions. You can email, call, or schedule a video conference meeting to get your questions answered. The program may even offer an informational session for prospective applicants where you can learn more about how to be a competitive applicant.

It’s essential to start early when applying to programs, as most summer REUs have applications open from fall to winter and notify students in the spring (for more information, see Rule 5). Find out what the application requires and map out all the elements. Generally, an application will ask for an essay of some sort, letter(s) of recommendation, transcripts, or other materials. Use Table A in S1 File, along with Table 1 below, to keep you organized throughout the process. This will help you build an application timeline and materials checklist while helping you stay organized.

**Table 1:** Use this planning table to stay organized while identifying potential REU programs.

**Program information (e.g., website, email, contact information)**	**Program dates**	**Application deadline and materials**	**Reference letter writers (e.g., trusted mentors, advisors, or professors who know you well and can speak on your potential)**	**Potential REU research mentors (e.g., a faculty member from the program’s website. Note some program will do that matching for you)**	**Stipend amount (e.g., how much money the program will pay you for the summer)**	**Program “Perks” and benefits (e.g., housing, meals, technology, accommodations, etc.)**	**Additional information (e.g., community support, transportation, etc.)**

We encourage you to share your planning table and research program list with a trusted mentor or peer who has experience with REUs. You can also strengthen your application materials by drawing on your network and asking trusted mentors and peers to read and provide feedback on your materials. If you don’t have a mentor, ask a professor or instructor from a class you enjoyed about what they recommend. They might be willing to offer support or guide you in the right direction. The application will likely ask for a letter of recommendation. Generally, you should ask someone who knows you and can speak to your potential. For in-depth guidance on the letter-seeking process, see 10 simple rules for navigating the reference letter seeking process [27] and 10 simple rules for writing compelling recommendation letters [28].

## Rule 5: Apply and wait and see if you get an offer

It’s time to shoot your shot! Let all your hard work from Rules 1 to 4 shine in your application. Reviewers will be looking for research fit, mentor–mentee match, and program interest. Applying to multiple programs will increase your chances if you maintain quality and intention throughout each application you submit. This includes tailoring your application specifically for each program you apply to and for each question they ask of you (instead of using the same general response for each prompt or essay). For example, ensure that all your application materials reflect the program you are applying to by adding specific reasons why you’re interested in conducting research at that particular institution. Double-check your application materials to avoid submitting the wrong application to an unintended program. Make sure your letter writers also know which programs they are submitting a reference letter to.

If you receive an offer, revisit your needs outlined in Rule 2 and in S1 File. Ask the program staff any follow-up questions regarding funding support, timeline, and expectations. This will help you make an informed decision as you consider your offer(s). If unforeseen barriers arise, do not hesitate to contact program staff or staff at your home institution to request additional help. Though not guaranteed, some programs can be creative with funding, so it doesn’t hurt to ask!

**Do not accept more than one program’s offer if you receive more than one acceptance**—if you are waiting to hear back from other programs, share this information with the REU staff who offered you a position. They recognize that competitive applicants may face multiple offers and can usually be flexible in their acceptance deadlines. Once you have accepted one program’s offer, follow up with the other offers you received to decline their acceptances.

It’s important to keep in mind that REU programs are extremely competitive, and you might not get accepted on the first try. If you are not accepted into a program, know there is no failure if you gain something from the application experience. Whether it is strengthening your scientific ideas, engaging with your STEM community, or learning more about yourself, there is a lot to build from that will be helpful for your development as a scientist. After all, iteration is essential in the scientific process—so don’t give up!

If you would like feedback on your application, reach out to your support networks, such as faculty advisors, trusted professors, or career counselors to discuss your materials. You may also consider inquiring with REU program staff, though many programs may not offer this type of personalized feedback. Your networks can offer guided feedback and help you craft a stronger, more refined application when you are ready to apply for future research opportunities.

## Rule 6: Set expectations with your research mentors—and yourself!

Once you accept the program’s offer, your program of choice will connect you with your faculty research mentor, also known as the principal investigator (PI), and their lab. This early connection can help you learn about current project goals and start conversations with mentors and lab mates. You will be working with this community of scientists throughout the program. Your primary research mentor(s) may be a graduate student (master’s or PhD student), a postdoctoral fellow, or a research staff scientist. Your primary research mentor(s) facilitate day-to-day research training and can help you understand the lab’s unique culture and research culture more broadly. You can also build relationships with others in the lab, even if you are not directly working on a project together. This tiered mentorship can provide near-peer mentoring and help expand your academic and personal network. For more information about how a lab is set up and the people who make up a lab group, see Dr. Kathy Baker’s book *At the Bench*: *A Laboratory Navigator* [29].

As you begin your research experience, keep in mind that building reciprocal relationships is vital to being a successful researcher and collaborator. The impact of mentorship with active and engaged mentors can help you cultivate your confidence and increase your sense of belonging in STEM. Remember that a scientific mentor is slightly different from a scientific advisor. We define a scientific mentor as a scientist, like a faculty research mentor, who has agreed to invest in your personal and professional growth and development, whereas an adviser shares scientific knowledge, provides feedback, and oversees the direction of your research. For more examples of how scientific mentors differ from advisors, see [30,31].

Recognizing the role your faculty mentor and your primary research mentor will play in your scientific growth and development is crucial. Discussing expectations and setting deadlines with your mentor(s) will help set the tone for your REU experience and demonstrate to them that you value their time and energy. Some norms to establish include how frequently you will communicate with one another and your preferred communication styles. Work together to set goals and understand how your work fits into the larger aims of the group. Use the Setting Research Expectations and Goals section of the REU planning packet to co-create research goals and understand each other’s expectations [16]. Then, revisit these expectations and deadlines throughout the summer to discuss what is going well and what can be improved.

While setting expectations with your research mentor(s), it’s essential to also set expectations with yourself. Be patient with yourself and ask for support when you need it. REUs are an opportunity for you to learn and further develop as a scientist, so don’t expect to know everything immediately. Failure is an integral part of the scientific process, so be kind to yourself and practice a growth mindset [32] when scientific failures arise. If you join a program that isn’t perfectly aligned with your research goals, think of ways to leverage the experience. Many REU programs may offer research seminars, graduate school preparation, and professional development workshops. Presenting your research at the end of the summer will also help develop your scientific communication skills and help expand your academic network. Use this experience to grow personally and professionally and clarify your long-term goals.

## Rule 7: Be Intentional about socializing in and out of the lab

Social ties are important for retention in STEM [33–35], and mentoring can come in many forms, including from people you meet outside the research lab. During the REU, build connections with others in your program and your research lab. REU programs may partner with offices like student clubs, writing centers, and student wellness programs to support your work-life balance during your REU. Use these connections to get to know people throughout the campus community. Reach out and ask people about their experiences in STEM, their career journey, and what the culture is like at their institution, including in their particular program. You can even ask your research mentor(s) to help connect you to current students, especially if you want to pursue a graduate or professional degree. Informal conversations can be just as illuminating as formal ones.

**Fig 1:**
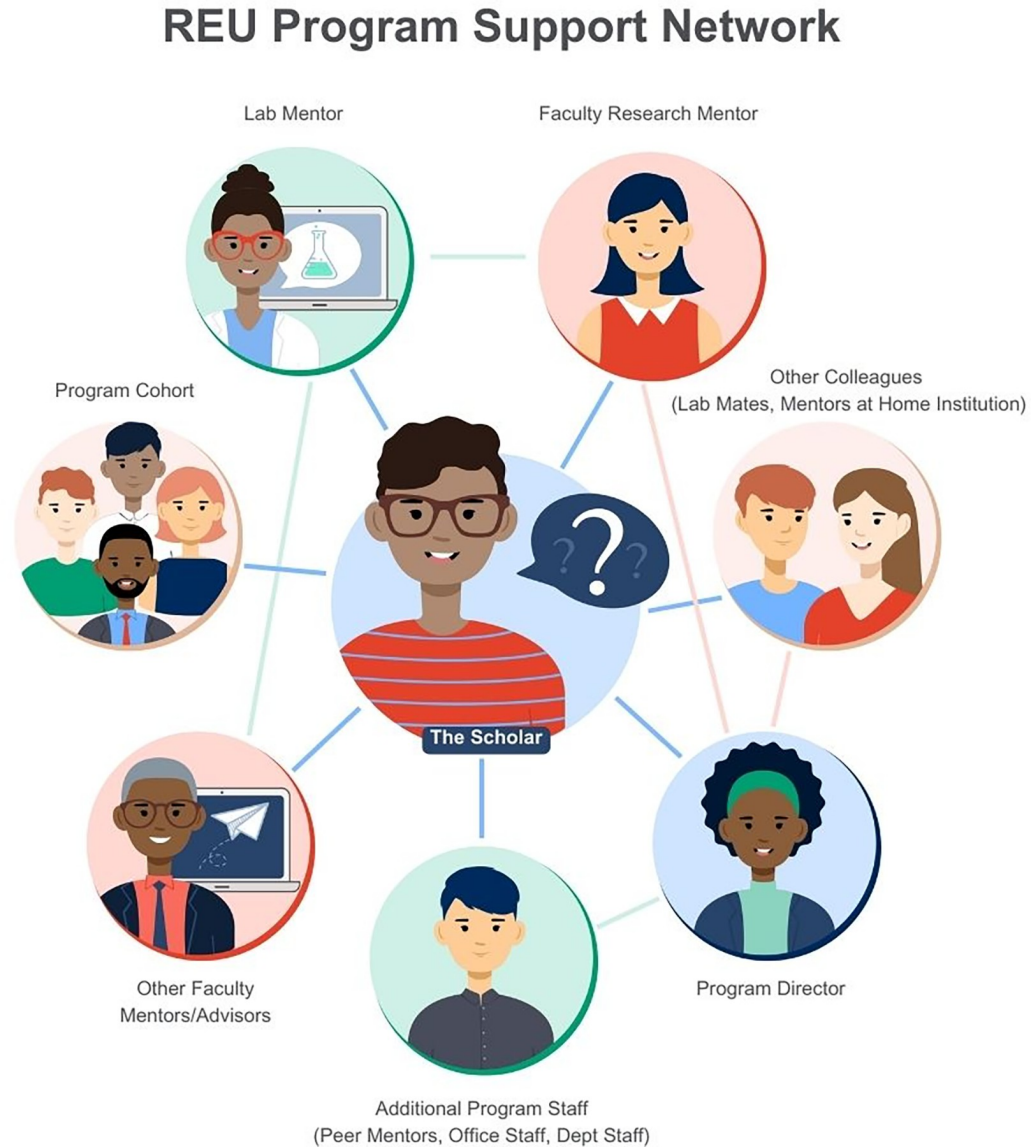
REU program support network map. It’s important to note that there are many people involved in the undergraduate research process—including your faculty mentor (often referred to as your “PI”) and the person in lab who will be your day-to-day contact (often a graduate student or postdoctoral researcher, referred to here as “Lab mentor”). When taking part in an REU program, you not only receive that in-lab support, but also have external support through the program director and other program staff, and even your cohort mates who are also taking part in the program. More broadly, you can seek support from mentors at your home institution and collaborators in other labs who work on similar research. This map aims to illustrate the support network that every REU student participant, referred to in the network map as “The Scholar,” has to help them succeed in their REU experience and beyond. Illustration developed by Gabriella Castillo.

Fig 1 shows the expansive support network that may be available to REU program participants, referred to as scholars. If done with intention, the connections you make during your REU can prove to be academically and socially fruitful in the future. For example, your faculty research mentor can be a reference letter writer for future opportunities, like applications for other REUs, graduate or professional programs (e.g., PhD or MD applications), or scientific funding opportunities [27,28].

## Rule 8: Grow and practice your scientific communication skills

An important part of being a scientist is communicating your ideas to others [36,37]. You can use your REU experience to practice your science communication skills (see [38] to learn more about building this skill). While conducting research, you will likely experience a shift in your understanding of science from being a “consumer” to a “producer.” As a student, you have been consuming science (i.e., attending lectures, reading textbooks, studying for quizzes/exams). As a researcher, you will produce science by designing and executing experiments, analyzing data, generating results, and seeking feedback on next steps. As a producer, you will be expected to convey the outcomes of your efforts to others. For example, your lab group may ask you to give research updates in weekly lab meetings or share your findings in an oral or poster presentation. These are important opportunities to practice your scientific communication skills. This can be intimidating at first, but there are helpful strategies that can help ease you into this new role.

As a producer of science, it’s important to understand your “why” in the project. As you conduct your research, practice the QUERY method to help deepen your understanding of your project, where QUERY is an acronym for “Question, Experiment, Results, and Your Interpretation” [39]. Use the QUERY method to answer questions like: What are your overarching research questions and hypotheses for this project? How does your research connect to the overarching question and what your lab group investigates? Ask yourself what you hope to learn and how this relates to the broader research community and your future career goals. Refer to the research expectations and goals (Rule 6) you created with your research mentor(s) and update the questions, hypotheses, and aims throughout your research experience, especially as you build out presentation materials. Discussing this with peers (formally and informally) will help you build your communication skills as you answer follow-up questions and receive feedback on expressing those ideas and findings more clearly.

## Rule 9: Empower yourself to ask questions and seek support

You are in a new environment and are expected to have questions. It can feel intimidating to ask questions, but now is the time to challenge yourself and seek the information you need to grow as an independent scientist. Growing as a scientist will require you to ask questions like: What does progress look like? What does it mean when your science isn’t working? You won’t have “glowing results” or “positive data” all the time. The nature of your project might not work, but that is okay! It’s all about iteration. If you are present and doing the work, you can learn from “failure,” which is essential in the science community and process.

Employ “mentoring up” techniques [40,41] by taking an active (not passive) role in your mentorship experience. In this practice, mentees “actively engage in and share responsibility for making the relationship beneficial for both mentor and mentee” [40]. You can “mentor up” by actively getting to know your mentor’s priorities and communication norms for the summer. Additionally, rather than expecting your mentor to do all the scheduling logistics, you can take initiative in setting up meetings, check-ins, and follow-ups. Doing this will demonstrate that you are an active participant in the mentor–mentee relationship and are willing to share the labor in your mentorship experience.

Self-advocacy is a skill you should continually practice in your scientific career and other aspects of your life. This is especially critical if you are a student who belongs to underrepresented or marginalized identity groups, given that the diversity of the STEM workforce is still evolving [42], and finding support is essential for your success. Though cultural awareness and responsiveness are integral to holistic mentoring practices, not all mentors practice them. You may also face systemic barriers which threaten to impact your success in STEM [18,43,44]. In these situations, it can be difficult knowing what type of support you need. Even if you don’t know what to ask for, you can reach out to your lab mentor, PI, and others in your support system who may have a deeper understanding of the issue you are facing. They can provide suggestions and help to brainstorm solutions.

## Rule 10: Enjoy the experience and wrap up your REU through reflection

Getting involved in science is exciting, so don’t forget to enjoy what you’re doing! This is a protected time for you to grow and learn as a scientist and as a whole person. Summer research opportunities are a great way to make new friends and allies across disciplines. These experiences can offer opportunities for you to live in a different city, explore local culture, and diversify your goals.

Reflect on your REU with your mentors and yourself as you progress through the program. What have you learned? How have you grown as a scientist? At the end of the program, think about what you liked and what you wished was different. Has this experience impacted your future career goals?

Closing the loop with everyone you met during your REU experience is also essential. You can have an exit conversation with your mentors and peers to think through your experience. Think about your “glows” (highlights from the REU), “grows” (opportunities to further develop as a scientist), and “thank yous” (whom you’d like to extend gratitude for helping you while you were in your REU) as you prepare to meet with your research mentors [43]. You can also discuss if you can present your research findings from your REU at undergraduate-focused conferences, like the Society for Advancement of Chicanos/Hispanics & Native Americans in Science National Diversity in STEM Conference (SACNAS) [45], the Annual Biomedical Research Conference for Minoritized Scientists (ABRCMS) [46], and the National Society of Black Engineers (NSBE) [47] convention (for more information, see [48]). Additionally, most research areas have disciplinary-specific conferences offered nationally or regionally. You should talk to your research mentor about what conferences might be applicable to you and the work you have done. Scholarships may be available from these conferences or external sources like Google Conference Scholarships [49]. Establish ways to stay connected post-experience and continue cultivating the relationship from afar. Ask people: how can we stay connected? Share your contact information (email or LinkedIn) and send a follow-up message after the summer ends to express gratitude for this unique experience.

Finally, now that you’ve completed your REU program, consider offering your support to other students who are navigating the process for the first time. You might be surprised by how much you know!

## Conclusion

REUs are a great way to explore scientific curiosity, define career goals and interests, and develop your own identity as a scientist. There are many benefits to this type of research experience and a lot to consider. Ultimately, you want to make this experience your own. Use the rules above and cultivate support systems with trusted mentors and peers to help you make the most of your summer REU program.

## Supporting information

S1 File REU program packet. The REU program packet is a valuable set of resources to help navigate the REU application process from exploring what research interests you, documenting necessary materials for the application, and tracking your research project goals and weekly commitments once your REU starts (including research experiments and programmatic requirements). Pages 1 through 4 can be used before applying to REU programs to brainstorm what you like about science, what research topics interest you, the strengths you can leverage in a new and unfamiliar environment, and why you are generally interested in research. Pages 5 and 6 can help you understand your programmatic needs and stay organized by documenting application materials and requirements in the provided timeline and checklist. Once accepted into an REU, pages 7 through 10 can be used with your primary research mentors, including your faculty research mentor (the principal investigator or PI for short), to co-create research expectations and research goals for the summer REU program. It also includes a section to discuss how you work best and what support you might need throughout the summer. You can use pages 7 through 10 a few weeks before the program starts or during the first week. After establishing your goals for the research project, pages 11 through 13 are a research timeline template you can use with your research mentors to help break down your research goals into weekly experiments and data analysis. The research timeline template is written for a ten-week program and can be modified based on your REU program length. It also includes a section to document program requirements, if applicable. Pre-filled text in the timeline template suggests tasks you could do to engage in your research project and discussion topics for you and your primary research mentors.
(PDF)
