# Supplementary material for: Ten simple rules for students navigating summer research experiences for undergraduates (REU) programs: From application to program completion
Source: PLoS Comput Biol. 2023 Nov 9;19(11):e1011573. doi: 10.1371/journal.pcbi.1011573 (PMC10635516; doi:10.1371/journal.pcbi.1011573)
Supplement: S1 File — The REU program packet is a valuable set of resources to help navigate the REU application process from exploring what research interests you, documenting necessary materials for the application, and tracking your research project goals and weekly commitments once your REU starts (including research experiments and programmatic requirements). Pages 1 through 4 can be used before applying to REU programs to brainstorm what you like about science, what research topics interest you, the strengths you can leverage in a new and unfamiliar environment, and why you are generally interested in research. Pages 5 and 6 can help you understand your programmatic needs and stay organized by documenting application materials and requirements in the provided timeline and checklist. Once accepted into an REU, pages 7 through 10 can be used with your primary research mentors, including your faculty research mentor (the principal investigator or PI for short), to co-create research expectations and research goals for the summer REU program. It also includes a section to discuss how you work best and what support you might need throughout the summer. You can use pages 7 through 10 a few weeks before the program starts or during the first week. After establishing your goals for the research project, pages 11 through 13 are a research timeline template you can use with your research mentors to help break down your research goals into weekly experiments and data analysis. The research timeline template is written for a ten-week program and can be modified based on your REU program length. It also includes a section to document program requirements, if applicable. Pre-filled text in the timeline template suggests tasks you could do to engage in your research project and discussion topics for you and your primary research mentors. (PDF) [file pcbi.1011573.s001.pdf]

REU Program Packet

Use this packet to help you grow in your scientific identity. This REU program packet includes ways for you to explore research opportunities, apply to REUs, and set research goals and expectations with yourself and your primary research mentors.

What Do I Like About Science?
What Are My Research Interests?

Beginning a research experience first requires understanding what areas of science you like or are interested in. Use the space below to reflect on what you like about science, your topics of interest, and why they interest you. You can refer back to these questions as your interests develop and change.

What excites me about science? Why do I want to do research?

What scientific topics capture my interest?
What scientific topics are less exciting or interesting to me?

These could be topics you have learned about in courses, topics you have heard or read about in the news, or based on your life experience (e.g., you know someone affected by a particular disease).

This handout is from "Ten Simple Rules for Students Navigating Summer Research Experiences for Undergraduates (REU) Programs: From Application to Program Completion" by Maria Manzanares, Courtney Peña, Kayla C. Kobak, and Miranda B. Stratton.

REU Program Packet

What Do I Like About Science?
What Are My Research Interests?

Beginning a research experience first requires understanding what science you like or are interested in. Use the space below to reflect on what you like about science, your topics of interest, and why they interest you. You can refer back to these questions as your interests develop and change.

What kind of impact do I want my research to have?
What have I enjoyed or not enjoyed about past research experiences?

How do I hope to grow as a scientist in the future?

REU Program Packet

Who Am I? What Are My Strengths?

Your identity as a scientist is important to grow and cultivate alongside the other social identities, values, and life experiences that make you uniquely you. Use the space below to explore what your journey has been like so far and how your strengths can help you in your scientific career.

Who am I? What are my values?
What life experiences have brought me here?

What strengths have I developed through my life experiences that can help me navigate new and unfamiliar situations?

REU Program Packet

Explore Why You're Interested in Research

Before applying to REUs, explore and reflect on your research interests. As you complete the chart below, ask your "whys" multiple times to help you. You might not know all your "whys," which is okay! Not every box needs to be completed; your reasons and "whys" might change over time, so start small.

Start with why you are interested in research. Then, answer why you want that. Repeat this process until you reach your core reasons. These reasons can help you determine what activities are your true step in the right direction rather than just doing something because you feel like you should.

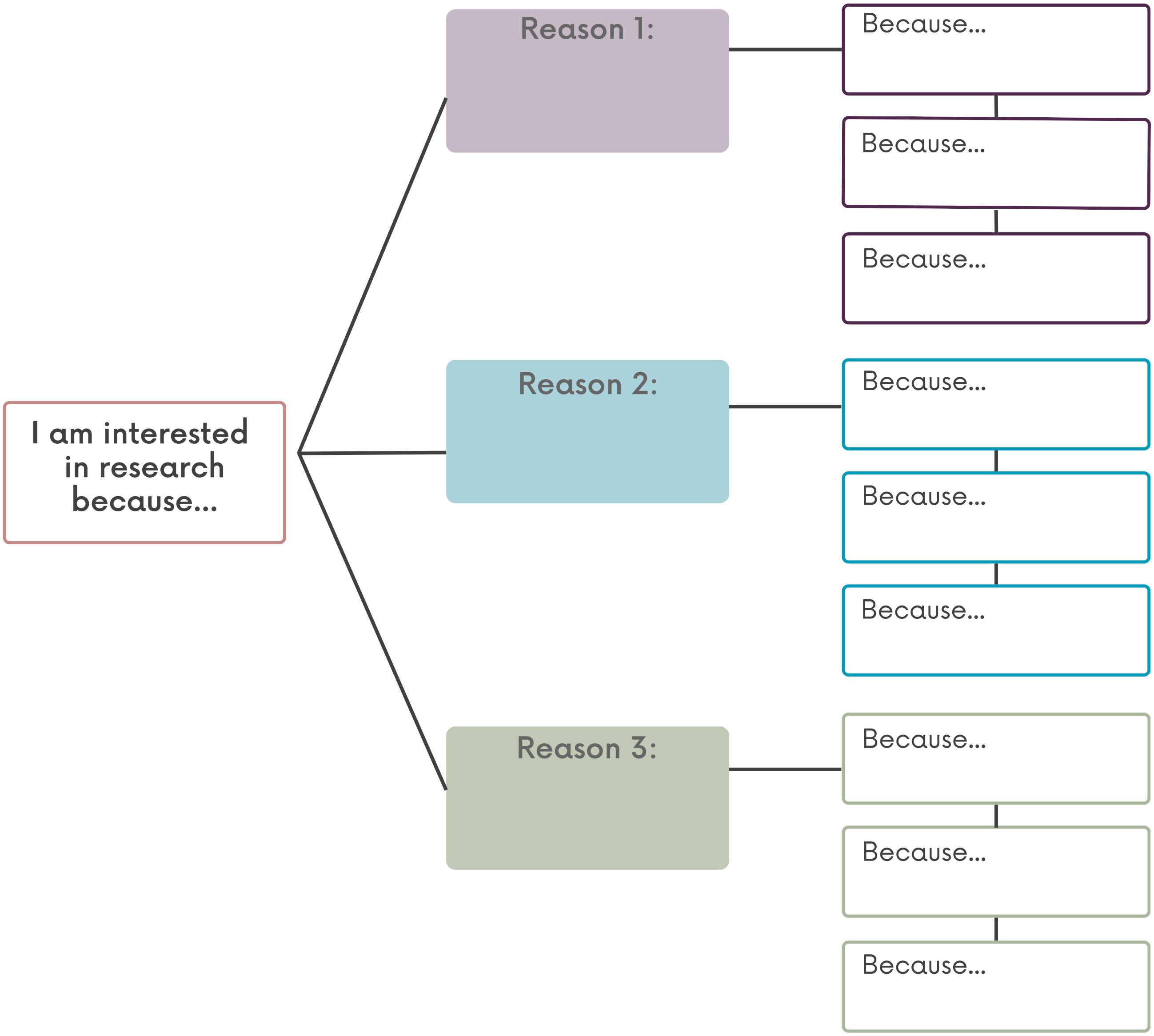

REU Program Packet

Identify Your Programmatic Needs

As you identify your programmatic needs, answer the following questions.

What is my availability, given my academic calendar?

What are my financial and housing needs?

Am I open to programs that are fully remote, hybrid, or in-person?

If pursuing a hybrid or in-person experience, will I need funding and logistical support for travel, housing, meals, or other needs?

If pursuing a remote experience, do I have the resources necessary to participate and reach my fullest potential (e.g., stable internet, a computer with high computing power, etc.)?

Can I leave my current living situation and shift my responsibilities to pursue an opportunity (e.g., apartment leases, job and/or family responsibilities, etc.)?

What are my accessibility-related needs (if any)?

What additional resources will I need to be successful?

REU Program Packet

Application Timeline and Materials Checklist

Table A. Use the table below to think through the requirements for each application.

Program Information (e.g., website, email, contact information)	Program Dates	Application Deadline & Materials	Reference Letter Writers	Potential REU Research Mentors	Stipend Amount	Program "Perks" and Benefits (e.g., housing, meals, technology, accommodations, etc.)	Additional Information (e.g., community support, transportation, etc.)

This handout is from "Ten Simple Rules for Students Navigating Summer Research Experiences for Undergraduates (REU) Programs: From Application to Program Completion" by Maria Manzanares, Courtney Peña, Kayla C. Kobak, and Miranda B. Stratton.

REU Program Packet

Setting Research Expectations and Goals

This section aims to assist research experience for undergraduates (REU) program students and research mentors, including faculty mentors, also known as principal investigators, in collaboratively setting research goals. This document promotes transparent, open, and honest communication among REU students and lab mentors. It can also be shared with program staff to track student progress. By co-creating research goals and openly sharing research expectations, we hope this will foster and strengthen mentoring relationships during the research experience. By establishing clear research expectations and communication among lab mentors and program staff, we hope it will lead to a productive and memorable experience for both students and mentors!

This template is designed for a ten-week summer research experience for undergraduates (REU) program with the expectation that students will conduct research at a full-time effort alongside program requirements. This template can be modified to best fit your REU program requirements, like research presentations. Reach out to program staff for the most accurate information so you can plan accordingly, including outlining and adapting weekly goals using the provided timeline.

Your Name:

Lab Mentor(s) Name(s):

Faculty Mentor (Principal Investigator, PI) Name:

Research Schedule (days & hours per week):

Primary Research Skills (including skills that will be developed this summer):

Primary Coding Language(s), if applicable:

Communication schedule with primary lab mentor(s):

Communication schedule with primary lab mentor(s) and faculty mentor:

REU Program Packet

Setting Research Expectations and Goals

Setting Research Expectations – REU Student

Please answer the questions below and discuss them with your lab mentor(s).

I would describe my learning style as....

Choose from the following - visual, auditory, kinesthetic (learning through movement), tactile (learning through touch), read and write

I work best when...

Include the time of day and frequency of communication with your primary lab mentor(s)

For a meaningful mentoring relationship, I would like my mentor to...

I will need guidance in...

Another thing to know about me is...

Setting Research Expectations – Primary Lab Mentor(s)

Please answer the questions below and discuss them with your REU student.

For a meaningful mentoring relationship, I expect my student to...

I would describe my mentoring style as...

I will make sure that I connect with my scholar...

Include frequency of communication (date/time) and format (email, phone call, Zoom, Slack, etc)

I work best with students when they...

Another thing to know about me is...

REU Program Packet

Setting Research Expectations and Goals

Setting Research Goals

For a successful summer experience, we encourage lab mentors to establish three research goals for their project: an **attainable goal**, an **expected goal**, and a **reach goal**.

Each goal can be phrased as a research aim or question to address throughout the project. The research project may already have these three goals in place; however, we suggest you and your lab mentor(s) review these goals together. We encourage you to seek feedback and modify the goals regularly as you grow in your scientific independence.

The "**attainable goal**" addresses the question - what will be the minimum achievable unit(s) that I, as the REU student, will be presenting on? This goal is important to remember if and when science goes poorly (e.g., datasets are unavailable from collaborators, etc.). The attainable goal(s) will ensure that you can present your findings at the end of your REU experience in a poster or presentation.

The "**expected goal**" is the intended research plan. This is the goal that you, as the REU student, should expect to reach if the project progresses steadily and according to plan.

The "**reach goal**" addresses the question - what are some potential "out of reach, or extraordinary goal(s)" of the project? This goal is extremely helpful if you have previous research experience and everything works the first time perfectly. Reach goals may also require a lot of luck, in addition to perseverance, as science is often fickle and unpredictable.

REU Program Packet

Setting Research Expectations and Goals

<u>Setting Research Goals</u>

Research Project - Brief Overview Description (3-5 sentences):

Overarching Research Question(s):

<u>Research Hypothesis and Goals:</u>

List the hypothesis or potential observations and results for this project:

List the “attainable goal(s)” for the research project:

List the “expected goal(s)” for the research project:

List the “reach goal(s)” for the research project:

REU Program Packet

Research Timeline

Research Timeline

A research timeline is useful in helping you, as the REU student, and lab mentor(s), to be aware of upcoming programmatic commitments. REU program staff should be contacted for the most recent updates, including assignments. Mapping these weekly commitments, including due dates, can help students and lab mentors co-create, plan, and adjust weekly research goals accordingly.

The following research timeline table is a template for a 10-week research experience. However, you can adapt this timeline to best fit your program's duration if it is shorter or longer than a 10-week research experience.

REU Program Packet

Research Timeline

Program Dates	Research Goal(s), including Technical Skills	Program Commitments, including days off or out of the lab
Prior to the start of the REU program	**Email introductions to scholar, lab mentor(s) and faculty mentor (principal investigator, PI)**<br>• Begin to develop the research plan and expectations<br>• Share pertinent papers related to the project	• Complete required trainings<br>• Meet to review project, research goals and expectations, communication preferences, and complete this document<br>• Download software for research and encrypt your computer (optional)<br>• Begin to read papers about the project
Week 1*<br>*Start of the REU program	**Start your research project!** **Continue to communicate with lab mentor and PI about project goals. Determine weekly meeting schedule with research mentor and PI**	• Begin research experiments & recording observations<br>• Read and discuss important papers related to the project<br>• Meet to discuss research goals and expectations in this template
Week 2	**Finalize research plan and expectations. If need be, share program goals with REU program staff for additional support**	
Week 3		
Week 4		
Week 5	**Revisit research plan (See Setting Research Expectations and Goals)**	

REU Program Packet

Research Timeline

Program Dates	Research Goal(s), including Technical Skills	Program Commitments, including days off or out of the lab
Week 6	Reassess your research project. Discuss a research back-up plan, or potential ways to pivot the project, if the intended project is not aligning with the expected goals	
Week 7	Pivot to the research back-up plan if the plan is not aligning with the expected goals	Discuss how to structure your research presentations and how to wrap-up your project
Week 8		Close out your research experience:<br>• Schedule exit conversation with lab mentor and PI on highlights, growth opportunities, and ways to stay connected post-experience
Week 9	If giving a talk or poster, the authors strongly encourage you to set up a practice talk with your research lab this week!	Close out your research experience:<br>• Create transition plan with your mentor(s) that includes information below
Week 10*<br>*End of the REU Program	Present research at the end-of-the program symposium, if required by the program.	Close out your research experience:<br>• Transfer data, code, lab notebooks, & additional lab information to lab mentor. Return any office/lab keys<br>• Return loaned materials to lab or program (if applicable)
Post-REU*<br>*ideally within four weeks of completing REU	Practice gratitude: Thank your mentor(s), PI, any faculty, or grad students you have connected with.	This is also a good time to thank others who have helped you, like the REU program staff. You can write a card or send a follow-up email after the REU ends. Don't feel obligated to give gifts - a kind note goes a long way!
